# Developmental changes in attention to faces and bodies in static and dynamic scenes

**DOI:** 10.3389/fpsyg.2014.00193

**Published:** 2014-03-06

**Authors:** Brenda M. Stoesz, Lorna S. Jakobson

**Affiliations:** Department of Psychology, University of ManitobaWinnipeg, MB, Canada

**Keywords:** attention, cognitive load, development, dynamic faces, eye-tracking, motion

## Abstract

Typically developing individuals show a strong visual preference for faces and face-like stimuli; however, this may come at the expense of attending to bodies or to other aspects of a scene. The primary goal of the present study was to provide additional insight into the development of attentional mechanisms that underlie perception of real people in naturalistic scenes. We examined the looking behaviors of typical children, adolescents, and young adults as they viewed static and dynamic scenes depicting one or more people. Overall, participants showed a bias to attend to faces more than on other parts of the scenes. Adding motion cues led to a reduction in the number, but an increase in the average duration of face fixations in single-character scenes. When multiple characters appeared in a scene, motion-related effects were attenuated and participants shifted their gaze from faces to bodies, or made off-screen glances. Children showed the largest effects related to the introduction of motion cues or additional characters, suggesting that they find dynamic faces difficult to process, and are especially prone to look away from faces when viewing complex social scenes—a strategy that could reduce the cognitive *and* the affective load imposed by having to divide one's attention between multiple faces. Our findings provide new insights into the typical development of social attention during natural scene viewing, and lay the foundation for future work examining gaze behaviors in typical and atypical development.

## Introduction

Typically developing individuals show a strong visual preference for faces and face-like stimuli (Valenza et al., 1996; Downing et al., 2004; Nummenmaa et al., 2006; Langton et al., 2008). This face preference is present within several hours of birth (Nelson, 2001), and throughout childhood (Elam et al., 2010), adolescence (Freeth et al., 2010), and adulthood (Bayliss and Tipper, 2005; Hershler and Hochstein, 2005). A tendency to attend to faces at the expense of attending to objects is particularly evident when facial expressions are ambiguous, or when the stimuli are more realistic (Land and Hayhoe, 2001) and social (Foulsham et al., 2010). This makes sense, as faces are a rich source of information that can help us to respond appropriately during social interactions (Domes et al., 2013).

Studies exploring developmental changes in our attention to faces have shown that young infants look longer at static than at dynamic faces. Indeed, infants up to 4 months of age have been shown to fixate on the static faces of a toy monkey (Brazelton et al., 1974), a manikin (Carpenter et al., 1970), and a doll (Field, 1979; Legerstee et al., 1987) for longer periods than the dynamic faces of their own mothers. Looking away from the mother does not appear to reflect passive disinterest. Rather, when they look away, infants show expressions indicative of concentration, as if they were engaging in *time-outs* from the previous looking period (Field, 1979). Taking these time-outs may reduce infants' cognitive load by providing them with more time to process the rich information conveyed by moving faces (Glenberg et al., 1998; Doherty-Sneddon et al., 2002). This would be beneficial as infants are naïve perceivers of the world, for whom the processing of most stimuli is challenging and effortful (Bahrick and Lickliter, 2012).

Infants may reduce their cognitive load by shifting their attention from a moving face toward a moving body. Evidence in support of this idea comes from the work showing that 5-month-olds can discriminate and remember repetitive actions (i.e., blowing bubbles, brushing hair, and brushing teeth) better than the faces of the people performing those actions (Bahrick et al., 2002). Like faces, bodies provide important social information but, because the movements typically occur at a grosser level, bodies may be less challenging for infants to process.

With increasing age, infants' periods of looking away from moving faces become shorter. For example, between 3 and 9 months of age, infants increase the amount of time they spend looking at the faces of talking cartoon characters depicted in complex dynamic scenes (Frank et al., 2009). The increased time spent looking at faces may reflect infants' growing understanding that faces are a significant source of social information (Frank et al., 2009, 2012), but it may also reflect the fact that they are becoming increasingly proficient at processing dynamic cues (e.g., Wattam-Bell, 1996; Braddick et al., 2003), and increasingly sensitive to intersensory redundancy (e.g., the match between speech sounds and moving mouths) (Bahrick and Lickliter, 2000). The fact that infants' attention to faces becomes especially marked when they are *listening* to a speaker (Smith et al., 2013; Tenenbaum et al., 2013) supports the view that they use visual cues (lip movements) to facilitate speech perception (e.g., Bristow et al., 2009), although their ability to integrate visual and auditory speech cues is not as strong as that of adults (Desjardins and Werker, 2004).

Several studies have examined children's attention to faces as they listen and respond to questions posed by adults (Doherty-Sneddon and Kent, 1996; Doherty-Sneddon et al., 2002; Doherty-Sneddon and Phelps, 2005). These studies suggest that, by 8 years of age, children (like adults; Glenberg et al., 1998) use gaze aversion to help them manage their cognitive load. Specifically, as the difficulty of the questions being posed increases, children look away from the speaker when the question is being posed, and when they are formulating and articulating their responses. This behavior is evident whether children are engaged in face-to-face interactions or are viewing a speaker via video-link (Doherty-Sneddon et al., 2002; Doherty-Sneddon and Phelps, 2005), and suggests that processing the moving face of the speaker requires cognitive resources. Children's tendency to engage in gaze aversion when being spoken to may explain why they show a significantly smaller McGurk effect (McGurk and MacDonald, 1976) than adolescents or young adults (Desjardins et al., 1997; Tremblay et al., 2007). The McGurk effect is an audiovisual illusion that occurs when an individual is presented with mismatched visual and auditory phonemes (e.g., *ba* and *ga*), but reports perceiving a third phoneme (e.g., *da*). Young children are less likely than older participants to experience the illusion, reporting instead the auditory phoneme that was presented (Tremblay et al., 2007)—a result that suggests they are not attending closely to dynamic facial cues.

To the best of our knowledge, there have been no studies examining children's gaze behaviors during *passive* viewing of naturalistic scenes (see Karatekin, 2007 for a review). This is unfortunate because adding task demands can lead to gaze behaviors that are quite different from those seen under passive viewing conditions (Smith and Mital, 2013), and age-related differences in task performance may obscure or alter age-related changes in deployment of attention (Scherf et al., 2007). While studies examining passive viewing in children are lacking, some research involving typical *adolescents* suggests that they fixate significantly longer on faces than on bodies or objects while viewing movie clips of social interactions (Klin et al., 2002), and while viewing static *and* dynamic scenes depicting single or multiple characters (Speer et al., 2007). These gaze behaviors differ from those made by adolescents with autism, who fixate longer on objects than on either faces or bodies (Klin et al., 2002), and who make shorter fixations on eye regions and longer fixations on bodies than typically-developing peers, particularly when viewing dynamic, multiple-character displays (Speer et al., 2007). Together, these results suggest that, whereas typical adolescents direct their attention toward moving faces during passive viewing of scenes, those with autism look away from faces—perhaps in an effort to reduce their cognitive load.

Recently, a number of authors have examined the question of how adults control their attention to faces during different tasks. Although they do make more fixations on faces than on bodies, adults' person detection is improved when the whole person (i.e., face *and* body) is visible in a scene (Bindemann et al., 2010). A similar effect has been reported for person identification, especially when the stimuli are moving—a result that supports the view that movement of *both* the face and the body are useful during the identification process (O'Toole et al., 2011; Pilz et al., 2011). Together, these findings suggest that, when the body is visible, introduction of dynamic cues may encourage adults to shift some of their attention from the face toward the body (O'Toole et al., 2011). Additional support for this idea comes from the finding that adults' analysis of facial expressions is affected by the presence of emotional body language (Hietanen and Leppänen, 2008), even when task demands encourage them to direct their attention toward faces (Meeren et al., 2005).

The current study was designed to fill a gap in the literature by exploring how our attention to faces changes as a function of age. Specifically, we asked whether introducing dynamic cues or changing the number of people in a scene would have different effects on passive viewing behaviors, depending on the viewer's age. This question is of interest given that children's cognitive resources and processing efficiency are reduced compared to adults (e.g., Hale, 1990; Miller and Vernon, 1997); as such, we expected that our scene manipulations would place greater cognitive demands on younger viewers.

Face processing abilities, such as identity extraction, improve dramatically between 4 and 11 years of age (e.g., Carey and Diamond, 1977; Ellis and Flin, 1990; Johnston and Ellis, 1995; Mondloch et al., 2003; Ge et al., 2008). For this reason, we chose to compare the gaze behaviors of children whose ages were near the middle of this range (6–8 year-olds) to those of adolescents (12–14 year-olds) and young adults. We analyzed the average number and duration of fixations made in particular areas of interest (AOI: faces, bodies, background) as participants passively viewed naturalistic scenes. These variables were of interest as past research suggests that reductions in the number of fixations and increases in average fixation length reflect increasing processing demands (Henderson, 2003; Smith and Mital, 2013) and/or reduced processing efficiency (Açık, 2010). We also measured the total time that viewers devoted to examining each AOI or glancing off-screen in each trial (dwell time). Dwell time algorithms combine time spent executing saccades and fixating within an AOI (Salvucci and Goldberg, 2000), and dwell time has been examined in other research to assess viewers' preferences for and attention to faces (e.g., Matsuda et al., 2013). We expected that children would find it more challenging than adults to process moving faces and multiple-character scenes, and thus be more likely to shift their attention away from faces in these conditions in an effort to reduce their cognitive load. Adolescents were expected to perform at near-adult levels. By breaking up the scenes into different AOIs, we were also able to determine if children were more likely than adults to redirect their attention from faces toward bodies, objects, or off-screen.

## Methods

### Participants

Eighty-eight individuals participated in this study. We tested 32 children aged 6.0–8.0 years (*M* = 6.7, *SD* = 0.6; 13 boys, 19 girls), 26 adolescents aged 12.1–13.8 years (*M* = 12.8, *SD* = 0.6; 12 boys, 14 girls), and 30 young adults aged 18.1–26.8 years (*M* = 20.1, *SD* = 2.0; 17 men, 13 women). Children and adolescents were recruited via word-of-mouth and via local schools from Winnipeg and Altona, Canada. Young adults were recruited through the psychology participant pool at the University of Manitoba, Winnipeg, Canada. All participants were native English speakers and had normal or corrected-to-normal visual acuity.

### Materials

The 24 stimuli in the eye-tracking experiment consisted of clips from several episodes of a television series (the *Andy Griffith Show*) that originally aired on the CBS from 1960 to 1968. As outlined below, scenes were carefully chosen to meet certain criteria. First, the situations depicted were “realistic” in the sense that they were ones that individuals might experience in everyday life, and they took place in recognizable settings, such as a grocery store, a workplace, or on the street. In addition, scenes not only contained one or more people, but objects that one might naturally find in such situations (e.g., groceries, telephone, or park bench). We extracted 12 4-s video clips. Six clips depicted a single character conversing with an off-camera character, and six clips depicted two or more characters engaged in a social interaction. All interactions were emotionally neutral. In all scenes, at least the upper half of characters' bodies were visible, to allow us to determine if viewers' attention was being drawn from a character's face toward his/her body, toward objects in the background, or off-screen. In all dynamic scenes, the primary motion cues came from nonrigid movements of the face and/or body of the character(s), the character(s) did not move into or out of the field of view, and the objects in the background were generally stationary. To create the static displays, we extracted one static image from each movie clip; as such, each static image depicted the same character(s) and objects present in the corresponding dynamic display. Thus, this experiment consisted of four conditions: (1) single-character-static, (2) multiple-characters-static, (3) single-character-dynamic, and (4) multiple-characters-dynamic, with each condition consisting of six trials. Stimulus size was standardized at 640 pixels (23.8° of visual angle) wide and 480 pixels (18.0° of visual angle) high. Photographs had a resolution of 72 pixels per inch and the video was shown at 29 frames per second. No soundtrack accompanied the stimuli.

### Procedure

The study protocol was approved by the Psychology/Sociology Research Ethics Board at the University of Manitoba. Adult participants and parents of each child/adolescent who participated in the study provided written informed consent. Children and adolescents also confirmed their assent. Participants were tested individually. Each participant was seated approximately 60 cm from the 17-inch computer screen of a Tobii 1750 binocular corneal-reflection eye-tracking system (0.5° precision, 50 Hz sample rate, 5 fps per second, 1280 × 1024 pixels resolution; Tobii Technology Inc., Fall Church, VA). Because this particular eye-tracking system compensates for large and rapid head movements, participants sat entirely unrestrained (i.e., did not wear helmets, chin-rests, or markers). Tobii Studio Enterprise experimental software controlled the stimulus presentation.

**Figure 1:**
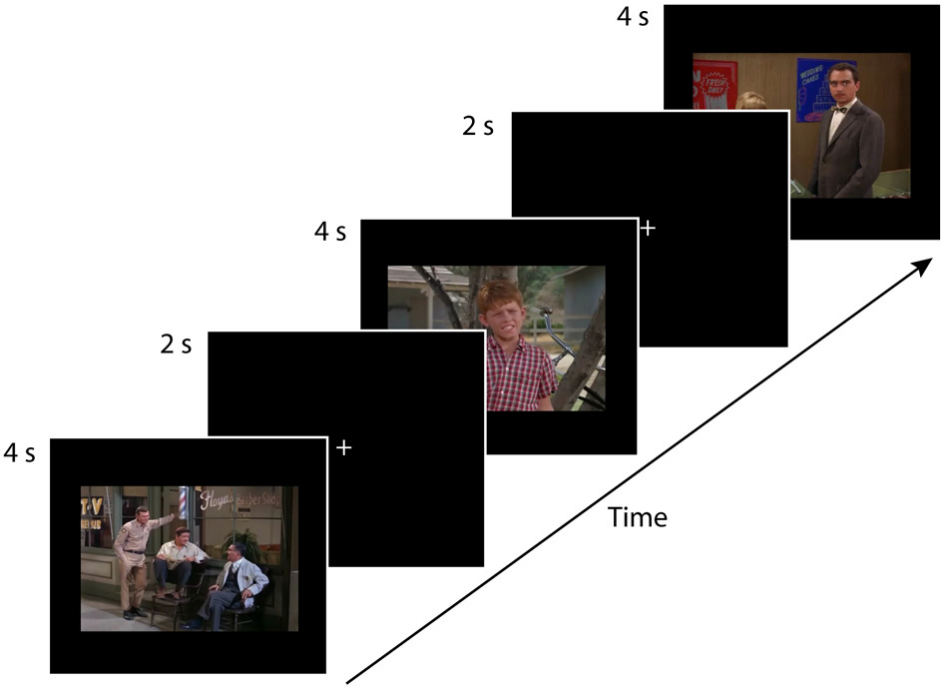
**This eye-tracking experiment consisted of four conditions**. (1) single-character-static, (2) multiple-characters-static, (3) single-character-dynamic, and (4) multiple-characters-dynamic, with each condition consisting of six trials. Each participant saw the 24 test trials in a random order. Each trial consisted of a 2-s central white fixation point on a black background followed by the presentation of the 4-s stimulus. The figure depicts three static trials (multiple-character, single-character, multiple-character). An example of a single-character-dynamic trial is provided in the supplementary material available online.

Before the experiment began, the experimenter carried out a short (approximately 15 s) 9-point calibration routine using the eye-tracker. Participants tracked a white dot moving on a black background. The dot moved slowly and randomly to nine locations on the screen. At each location, the dot appeared to grow and then shrink in size before moving to the next location. Upon completion of the calibration trial, Tobii Studio Enterprise experimental software gave immediate feedback regarding the quality of the calibration. The calibration routine was repeated if the quality was poor initially. Participants then engaged in free-viewing task consisting of 24 trials. Each of the 24 trials consisted of a 2-s central white fixation point presented on a black background, followed by the presentation of the 4-s stimulus (see Figure 1). Trials were presented in a different random order for each participant. The experiment took approximately 2.4 min to complete. Participants were instructed to look at the fixation cross at the beginning of each trial, and then to passively view each of the 12 photographs and 12 movies that would be presented one at a time.

### Analyses

Three areas of interest (AOI) were investigated: the face (or faces), the body (or bodies), and the background. Because the characters did not move rigidly across the screen in the dynamic scene, a complete a frame-by-frame analysis was unnecessary. This also made it possible to make AOIs of identical sizes in static and dynamic displays. Due to differences in camera viewing angle, the area of individual face AOIs were smaller in multiple- compared to single-character scenes. We ensured, however, that the total (combined) area of all visible face AOIs did not differ by scene type [single character: *M* = 3.91% of the scene, *SD* = 2.06; multiple-character: *M* = 2.68% of the scene, *SD* = 1.07; *t*_(10) = 1.31_, *p* = 0.22]. The total area devoted to body AOIs was also comparable in both types of scenes [single-character: *M* = 18.04% of the scene, *SD* = 6.88; multiple-character: *M* = 23.58% of the scene, *SD* = 5.47; *t*_(10)_ = 1.54, *p* = 0.15], as was the total area of the background [single-character: *M* = 78.04% of the scene, *SD* = 8.69; multiple-character: *M* = 73.75% of the scene, *SD* = 6.12, *t*_(10)_ = 0.99, *p* = 0.35]. Regardless of modality (static/dynamic) or scene type, the face AOI was smaller than the body AOI, which was smaller than the background AOI [*t*_(5)_ > 6.57, *p* < 0.001, in all cases].

Using Tobii Studio Enterprise software, we extracted a series of measures of gaze behavior with each AOI during each trial. The first was the number of fixations made within the AOI. Fixations were defined as any period where gaze stayed within a 30 pixel (0.9° of visual angle) diameter area for 200 ms or more. The second measure of gaze behavior was mean fixation duration. Our third measure was dwell time, which refers to the total time from the onset of the first fixation inside the AOI to onset of the first fixation outside the AOI. For the dwell time variable, we also calculated the amount of time that participants did not look at the screen by subtracting the total dwell time within the pre-defined AOIs from the total time each stimulus was on the screen (4 s). Finally, we computed the average for each variable across the six scenes within a condition.

The mean number of fixations and the mean fixation durations were entered into two separate 3 (Age Group: children, adolescents, young adults) × 2 (Scene Type: single-character, multiple-character) × 2 (Presentation Mode: static, dynamic) × 3 (AOI: faces, bodies, background) analysis of variance tests (ANOVAs), with repeated measures on the last three factors. Dwell time data were entered into a 3 (Age Group: children, adolescents, young adults) × 2 (Scene Type: single-character, multiple-character) × 2 (Presentation Mode: static, dynamic) × 4 (AOI: faces, bodies, background, off-screen) ANOVAs, with repeated measures on the last three factors. Variance assumptions for all comparisons were tested with Levene's test of equality of variances. Where violations of sphericity were observed, within-group effects were reported with Greenhouse-Geisser corrections. Follow-up multiple comparison tests on significant interactions were completed using Fisher's LSD tests. We analyzed the data using SPSS 22 (SPSS Inc., Chicago, IL, USA). Note that, before running the ANOVAs we confirmed that age was not related to scores on any of the dependent variables in the sample of young adults. This step was deemed necessary because the age range in the adult group was larger than the age ranges in the other groups.

## Results

### Number of fixations

**Figure 2:**
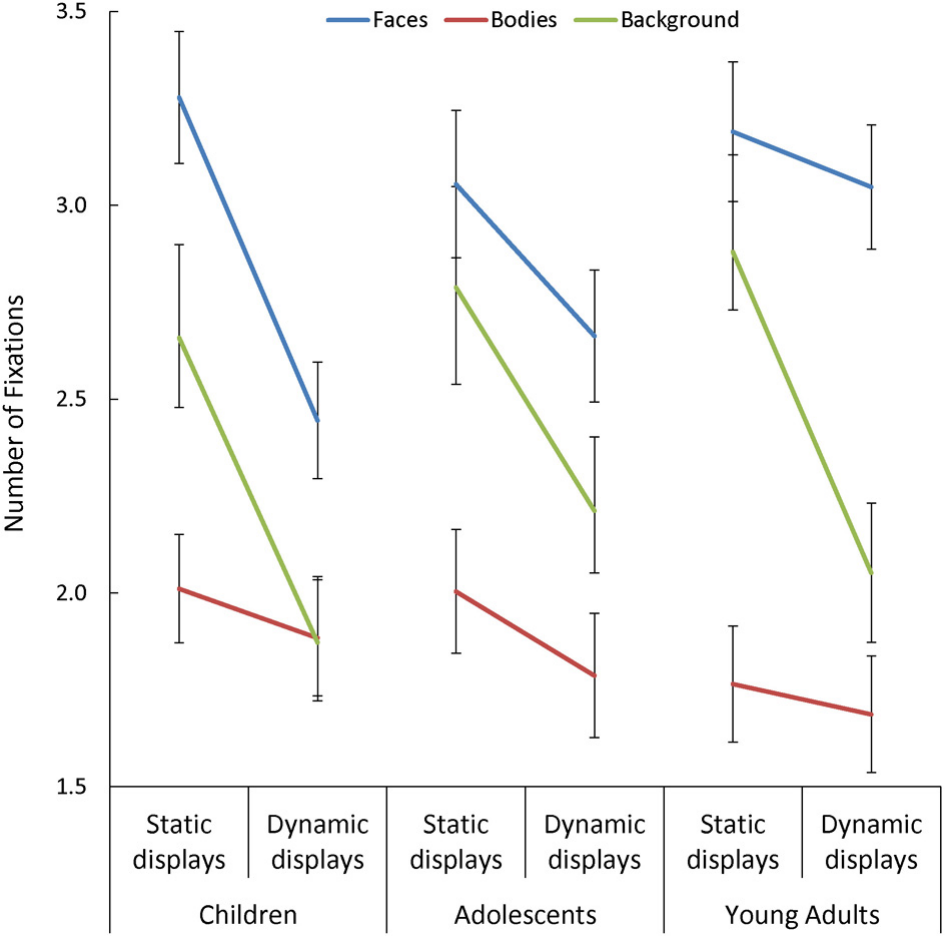
**The number of fixations made by children, adolescents, and young adults within the each area of interest (AOI; faces, bodies, and backgrounds) for the static and dynamic scenes**.

Overall, participants made more fixations within face AOIs, and fewer within body AOIs, than in the background [*F*_(1, 170)_ = 36.41, *p* < 0.001, η^2^_*p*_ = 0.30]. Participants also made fewer fixations when viewing dynamic than static scenes [*F*_(1, 85)_ = 114.38, *p* < 0.001, η^2^_*p*_ = 0.57], but this effect was: (a) larger in children than in adults [Presentation Mode × Age Group: *F*_(2, 85)_ = 3.13, *p* = 0.049, η^2^_*p*_ = 0.07]; and (b) most pronounced in the background [Presentation Mode × AOI: *F*_(2, 170)_ = 16.62, *p* < 0.001, η^2^_*p*_ = 0.16]. In addition, we observed a significant three-way interaction between Presentation Mode, AOI, and Age Group [*F*_(4, 170)_ = 3.30, *p* = 0.02, η^2^_*p*_ = 0.07; see Figure 2], and follow-up tests on this interaction revealed important age-related differences in the effect that adding dynamic cues had on the number of fixations made in face AOIs, specifically. On average, participants in all three groups made a similar number of fixations on faces during static trials but, as predicted, children and adolescents showed a significant drop in the number of fixations made in this AOI with the addition of dynamic cues [*t* > 3.44, *p* < 0.003, *d* = 0.67], whereas adults did not. This resulted in children making significantly fewer fixations on dynamic faces than adults [*t*_(60)_ = 2.80, *p* = 0.007, *d* = 0.71].

**Figure 3:**
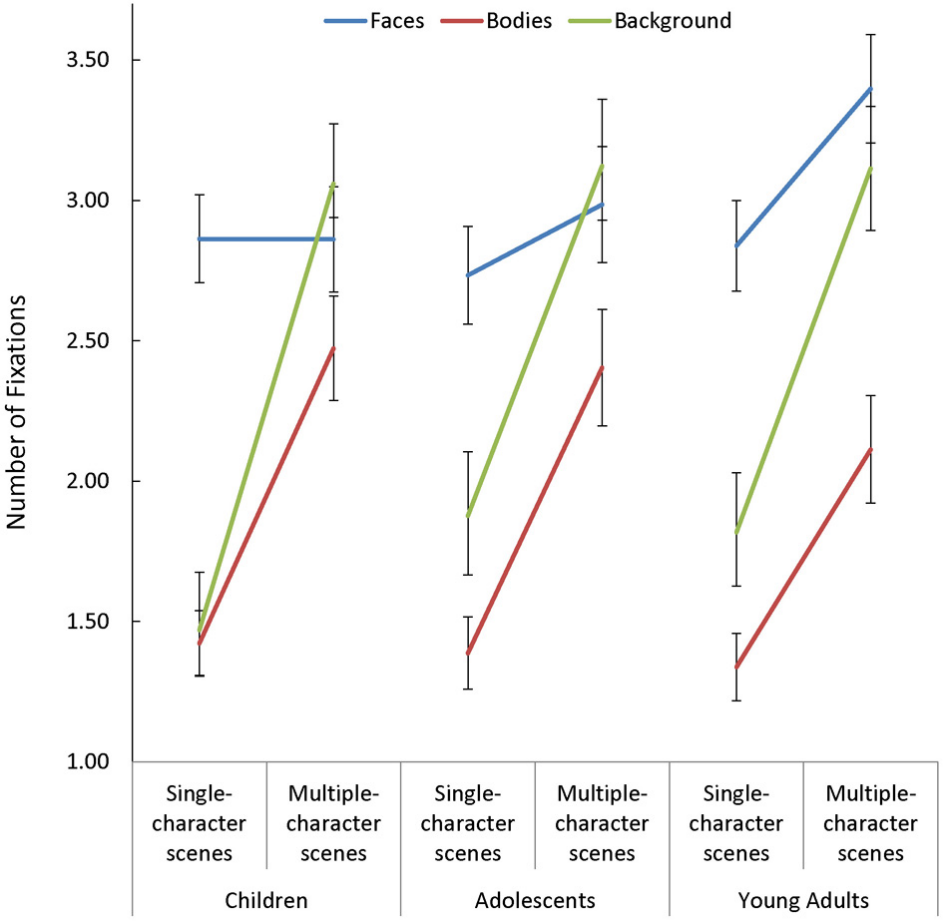
**The number of fixations made by children, adolescents, and young adults within the each area of interest (AOI; faces, bodies, and backgrounds) for single- and multiple-character scenes**.

In addition to the above, participants made more fixations when viewing multiple- compared to single-character scenes [*F*_(1, 85)_ = 317.431, *p* < 0.001, η^2^_*p*_ = 0.79]. While this Scene Type effect was smaller in face AOIs than in other regions [Scene Type × AOI: *F*_(2, 170)_ = 32.12, *p* < 0.001, η^2^_*p*_ = 0.27], the impact of changing scene type on the number of fixations made on *faces* varied as a function of age [Scene Type × AOI × Age Group: *F*_(4, 170)_ = 2.44, *p* = 0.049, η^2^_*p*_ = 0.05]. Specifically, as seen in Figure 3, adults increased the number of fixations they made on faces when additional characters were added to a scene [*t*_(29)_ = 2.86, *p* = 0.008, *d* = 0.52], but children and adolescents did not.

### Fixation duration

**Figure 4:**
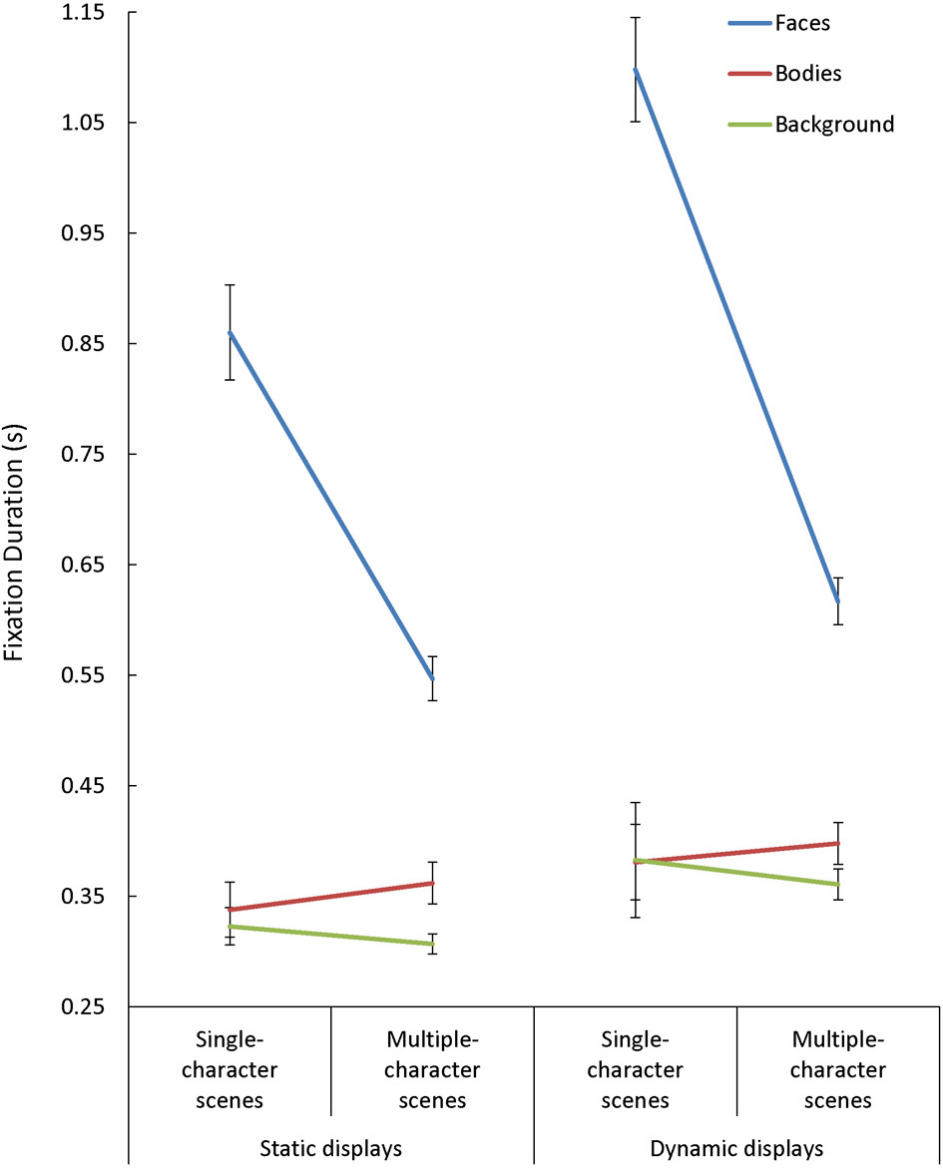
**The mean fixation duration(s) for each area of interest (AOI; faces, bodies, backgrounds) while participants viewed static and dynamic displays in single- and multiple-character scenes**.

Overall, mean fixation duration was longer during viewing of dynamic compared to static scenes [*F*_(1, 85)_ = 40.15, *p* < 0.001, η^2^_*p*_ = 0.32], and shorter during viewing of multiple- compared to single-character scenes [*F*_(2, 85)_ = 83.04, *p* < 0.001, η^2^_*p*_ = 0.49]. Fixations made in face AOIs were also generally longer than those made in other regions [*F*_(2, 170)_ = 187.26, *p* < 0.001, η^2^_*p*_ = 0.69]. We observed Scene Type x Presentation Mode [*F*_(1, 85) = 4.44_, *p* = 0.04, η^2^_*p*_ = 0.05], Presentation Mode × AOI [*F*_(2, 170)_ = 10.03, *p* < 0.001, η^2^_*p*_ = 0.11], and Scene Type × AOI [*F*_(2, 170)_ = 56.31, *p* < 0.001, η^2^_*p*_ = 0.40] interactions, but each of these interactions needed to be interpreted in light of a significant 3-way interaction involving Scene Type, Presentation Mode, and AOI [*F*_(2, 170)_ = 3.30, *p* = 0.02, η^2^_*p*_ = 0.07] (see Figure 4). Follow-up tests performed on the interactions revealed two key findings. First, although mean fixation duration increased with the introduction of dynamic cues across AOIs [*t*_(87)_ > 2.40, *p* < 0.02, *d* > 0.25, in each case], this effect was largest for fixations made within face AOIs in single-character scenes. Second, the drop in mean fixation length seen with the introduction of additional characters was *only* evident in face AOIs [*t*_(87)_ = 8.23, *p* < 0.001, *d* = 0.88].

**Figure 5:**
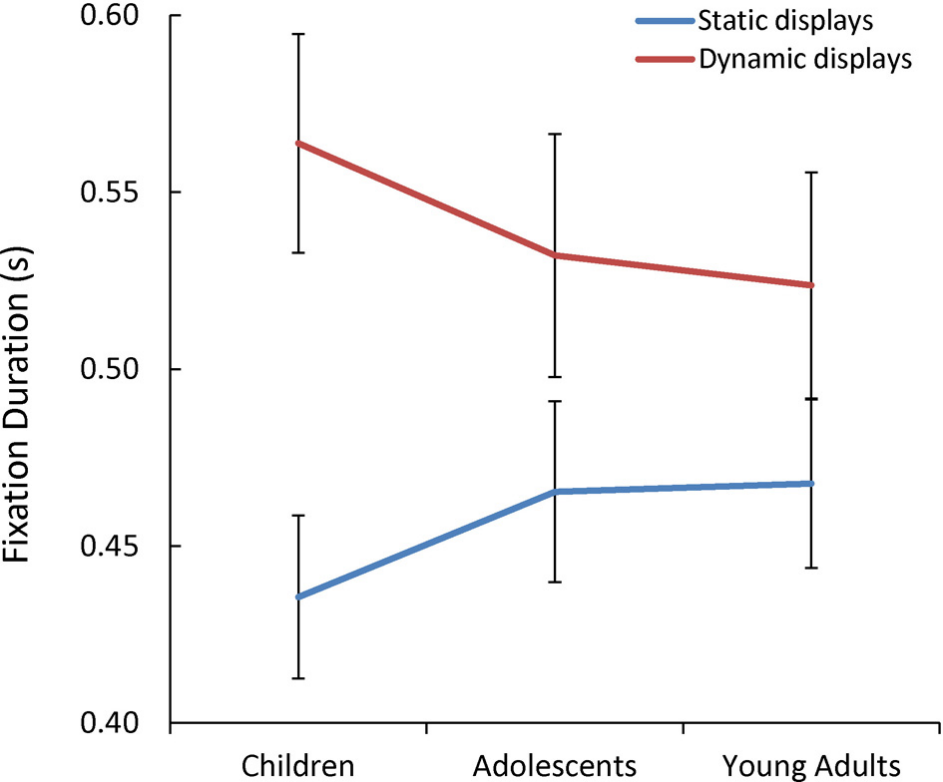
**The mean fixation duration(s) while children, adolescents, and young adults viewed each static and dynamic display**.

Additionally, we observed a significant Presentation Mode x Age Group interaction [*F*_(1, 85)_ = 3.07, *p* = 0.05, η^2^_*p*_ = 0.07] (see Figure 5). As predicted, only children's mean fixation duration increased significantly with the addition of dynamic cues [*t*_(60)_ = 2.09, *p* = 0.04, *d* = 0.53].

### Dwell time

**Figure 6:**
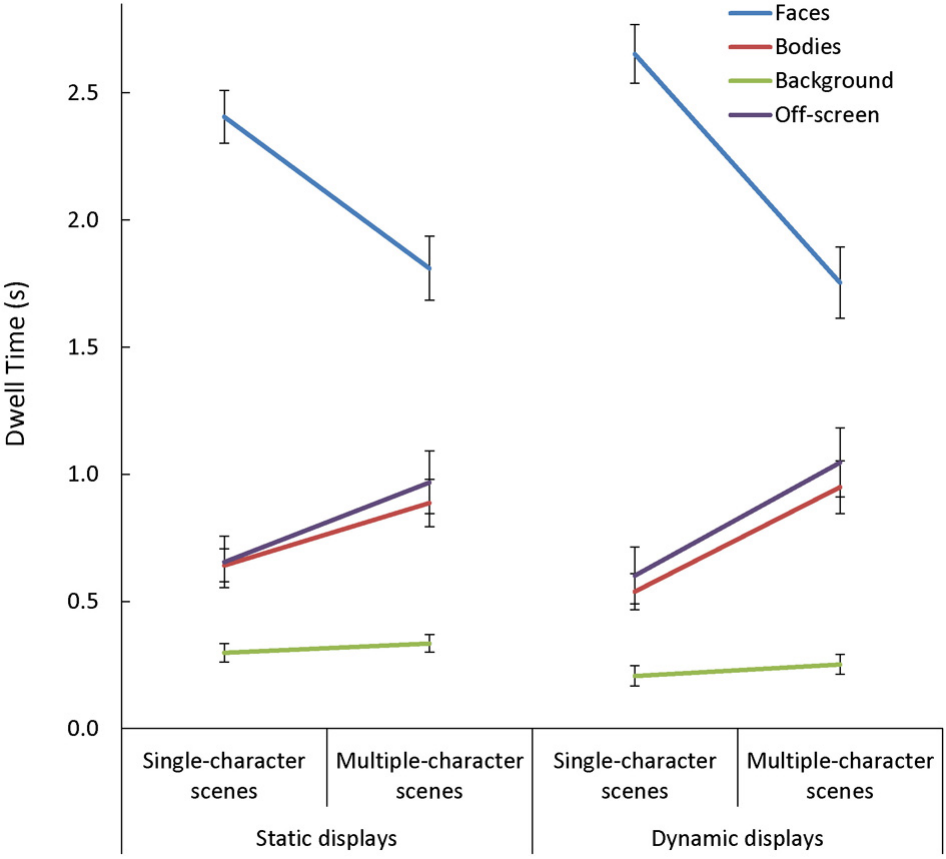
**The mean dwell time (s) in each area of interest (AOI: faces, bodies, backgrounds) and off-screen while participants viewed static and dynamic, single- and multiple-character scenes**. The Scene Type × AOI interaction seen with static scenes was amplified with the addition of dynamic cues. This was primarily due to the fact that viewers were more drawn to examine moving faces in single-than in multiple-character scenes.

**Figure 7:**
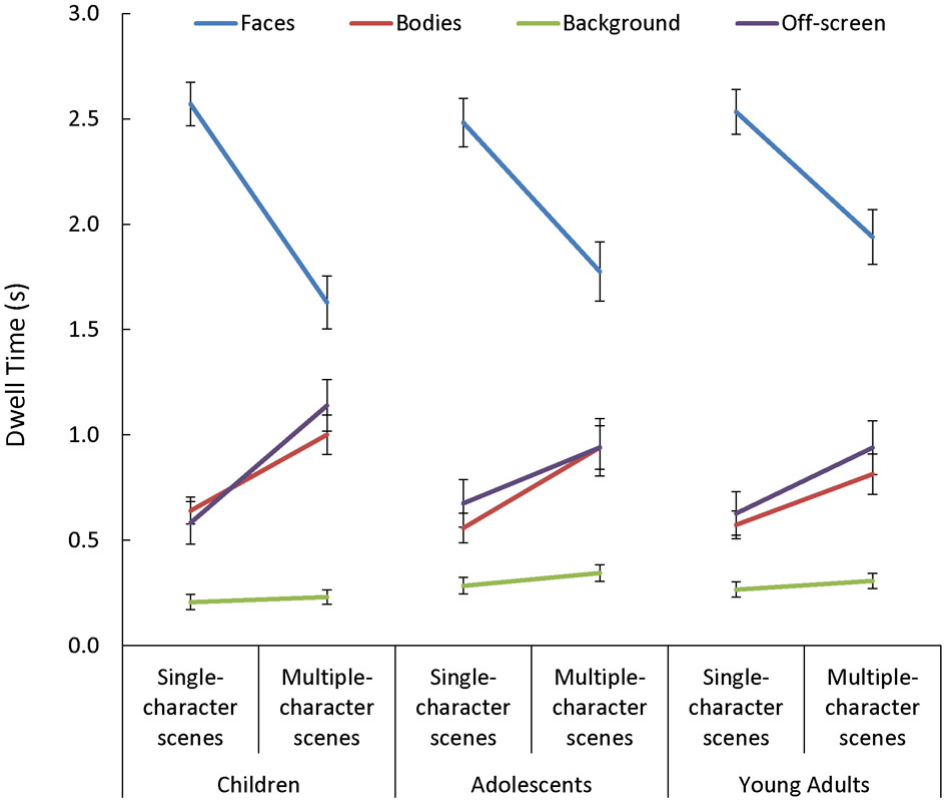
**Age-related changes in mean dwell time (s) in each area of interest (AOI; faces, bodies, backgrounds) and off-screen, for both single- and multiple-character scenes**. Although adding more characters to the scene triggered all participants to shift their attention from faces to bodies or off screen, these effects were larger in children than in adults.

In general, viewers spent more time looking at faces, and less time looking at backgrounds, than they did looking at bodies or off-screen [main effect of AOI: *F*_(3, 255)_ = 198.32, *p* < 0.001, η^2^_*p*_ = 0.70]. This main effect varied depending on the number of characters in the scene [Scene Type × AOI: *F*_(3, 255)_ = 84.13, *p* < 0.001, η^2^_*p*_ = 0.50]. Specifically, while the effect of AOI was present in both single- and multiple-character scenes [*t*_(87) > 3.62_, *p* < 0.001, *d* > 0.70, for all comparisons], adding more characters to a scene triggered participants to look less at faces [*t*_(87)_ = 11.91, *p* < 0.001, *d* = 1.27] and more at bodies or off-screen [*t*_(87)_ > 6.75, *p* < 0.001, *d* = 0.72, in both cases]. The Scene Type × AOI interaction was amplified when dynamic cues were added [Scene Type × AOI × Presentation Mode: *F*_(3, 255)_ = 9.06, *p* < 0.001, η^2^_*p*_ = 0.10; see Figure 6]. This was primarily due to the finding that viewers were more drawn to examine moving than static faces in single- than in multiple-character scenes [*t*_(87)_ = 5.03, *p* < 0.001, *d* = 0.54]. The Scene Type × AOI interaction also varied as a function of viewers' age [Scene Type × AOI × Age Group: *F*_(6, 255)_ = 2.25, *p* = 0.04, η^2^_*p*_ = 0.05, see Figure 7]. Specifically, although adding more characters to the scene triggered all participants to shift their attention from faces to bodies or off-screen, these effects were more dramatic in children than in adults [*t*_(60)_ > 2.52, *p* < 0.02, *d* > 0.63 for all comparisons].

## Discussion

The goal of the present study was to extend research on developmental changes in attention to faces by comparing the gaze behaviors of children, adolescents, and young adults as they viewed naturalistic scenes. We examined whether passive viewing behaviors in each age group would be affected by the introduction of motion and/or additional characters in scenes. We expected that each of these manipulations would make it more challenging for children, in particular, to attend to or process faces and that this would lead them try to reduce their cognitive load by engaging in more “looking away” behavior. In general, the results from the analyses of the eye-tracking data support these hypotheses. We discuss the findings below.

Despite the fact that the face AOIs were considerably smaller than any other regions, participants made more and longer fixations on faces than on other parts of the displays, which resulted in longer dwell times for faces. These results are consistent with recent eye-tracking studies (Birmingham et al., 2008; Bindemann et al., 2010; Rice et al., 2013) and other work showing that viewers of all ages are generally biased to attend to faces (e.g., Valenza et al., 1996; Downing et al., 2004; Nummenmaa et al., 2006; Langton et al., 2008). Although viewers may have focused on faces because there was little or no movement occurring in the background to capture their attention, this would not explain why we found the face bias during viewing of static, as well as dynamic stimuli. A more likely explanation of the face bias is that faces automatically attract attention due to their high social significance (see Lavie et al., 2003). As outlined below, however, factors such as the number of characters in a scene influence the way in which we divide our attention between faces and bodies. Additional insights into how we control our attention to faces when bodies are visible in a scene come from recent work on person detection (Bindemann et al., 2010) and person identification (O'Toole et al., 2011; Pilz et al., 2011).

Adding dynamic cues resulted in changes in participants' looking behaviors. In general, the addition of motion cues led to a reduction in the number of fixations and an increase in average fixation duration, but both of these effects were larger in children than in adults. As these effects are believed to reflect increasing processing demands (Henderson, 2003; Smith and Mital, 2013) and/or reduced processing efficiency (Açık, 2010), the present findings are consistent with the view that dynamic faces are more challenging for children than for adults to process. Children may also find dynamic faces more physiologically arousing, even when (as in the present study) the scenes are emotionally neutral. Interestingly, infants' arousal levels go down when their mothers slow down, simplify, or infantize their behaviors during interactions (Tronick et al., 1978)—a result that supports the view that face processing is both cognitively and affectively arousing for young viewers. In future work it might be interesting to vary the “affective load” across scenes, and look for age-related differences in phasic changes in heart rate and respiration amplitude, and in passive gaze behaviors.

As with the addition of motion cues, adding characters to a scene resulted in several changes in participants' gaze behavior. First, adults (but not children or adolescents) made more fixations on faces when viewing multiple-character scenes. This was true despite the fact that, in order to match the total area of particular AOIs across scene types, individual faces were smaller in multiple- than in single-character scenes. Second, adding characters to a scene led viewers in all age groups to decrease the mean duration of face fixations. Together, these results may reflect a competitive push-pull interaction between two sources of social information (see Findlay and Walker, 1999). Specifically, when attending to multiple characters in a scene, a viewer's eyes may be pulled from one face to another, resulting in more frequent, but shorter fixations on faces. It is also possible that our participants made shorter fixations on faces in multiple-character scenes simply because the individual faces were smaller, and therefore harder to resolve. In a related study, which involved static stimuli only, Birmingham et al. (2008) found that their adult viewers made *longer* fixations on the eye region of characters' faces as the number of people in the scene increased. In this study, actors were photographed from a standard distance, which meant that the total AOI for eye or face regions in multiple-character scenes was much larger than the area of the corresponding AOI in single-character scenes. It is important to note, however, that exposure durations were also much longer in the Birmingham et al. study than in the present investigation (15 vs. 4 s per trial). This may also have contributed to differences in the findings.

One strength of the current study is that we measured dwell times not just within particular AOIs, but also for off-screen glances. This proved to be important as these glances accounted for approximately 25% of total viewing times. Adding more characters to a scene resulted in viewers spending relatively less time attending to faces, and relatively more time attending to bodies or glancing off-screen. As one might expect if viewers found dynamic faces particularly difficult to process, these attentional shifts were especially evident when the characters were moving. In addition, shifts in attention from faces to bodies or off-screen were more pronounced in young children—supporting the view that children use gaze shifts like these to reduce their cognitive load. In future work, it would be interesting to study the effect that adding the soundtrack would have on viewers' gaze behaviors. This manipulation should increase the cognitive demands even further and, therefore, have a larger effect on children's than adults' gaze behaviors (see Doherty-Sneddon and Kent, 1996; Doherty-Sneddon et al., 2002; Doherty-Sneddon and Phelps, 2005).

Conducting studies with *information rich* displays that closely approximate naturalistic stimuli should be an important priority for researchers interested in face processing, as much of the existing literature in this area has utilized static displays. Studies incorporating moving faces or whole bodies—viewed in isolation or in the context of real-world scenes—are providing new insights into how we process social information (e.g., O'Toole et al., 2011; Pilz et al., 2011; Stoesz and Jakobson, 2013). In our lab, for example, we have used a Garner interference paradigm (Garner, 1976) to study how interference between the processing of facial identity and facial expression changes with the introduction of dynamic cues (Stoesz and Jakobson, 2013). We replicated earlier findings of bidirectional interference between the processing of these cues with static faces as in Ganel and Goshen-Gottstein (2004), and then went on to show that interference dropped to negligible levels when moving faces were used as test stimuli—results that suggest that viewers are better able to attend selectively to relevant facial cues when faces are moving than when they are static.

Like behavioral studies, most neuroimaging studies have investigated brain regions involved in face processing using static images, but this is beginning to change. Researchers have found that the visual processing of faces from static and dynamic displays involve different neural networks and/or different levels of activation of the same brain regions (Fox et al., 2009; Schultz and Pilz, 2009; Sato et al., 2010; Arsalidou et al., 2011; Kessler et al., 2011). Observations such as these lend weight to the suggestion that there is much to be gained from utilizing naturalistic, dynamic stimuli that are socially rich (see also Birmingham and Kingstone, 2009).

Exploring looking behaviors provides information on how components of our attentional system operate and what social interests we may have (Klin et al., 2002; Speer et al., 2007). Using eye-tracking technology to study eye movements and fixations has proven particularly useful for determining typical gaze behaviors in infants and adults, and contrasting these with gaze behaviors in various clinical groups. Our study makes a unique contribution to the literature on social attention, and is one of the first to examine gaze behaviors in three different age groups of participants—children, adolescents, and adults—during passive scene perception. The results are significant in that they provide additional insights into age-related changes in the deployment of social attention in response to changing cognitive demands associated with the introduction of dynamic cues, or additional characters. This work also provides a foundation for future studies we are planning that involve children born prematurely at very low birth weight (<1500 g). This group is known to be at risk for deficits in social perception and cognition. We have shown, for example, that children born preterm show impairments in their ability to use nonverbal face and body cues to interpret the emotions of people engaged in naturalistic social situations (Williamson and Jakobson, 2014). Incorporating eye-tracking in studies of this sort could help to determine if these deficits are associated with motion-processing problems and/or with gaze aversion or other atypical gaze behaviors. Knowing this may inform the development of interventions designed to improve social functioning in this at-risk population. Studies of this kind will also improve our understanding of the typical and atypical development of the social brain.

### Conflict of interest statement

The authors declare that the research was conducted in the absence of any commercial or financial relationships that could be construed as a potential conflict of interest.
